# PREDICTORS OF ANXIETY SYMPTOMS AMONG OLDER ADULTS WITH COGNITIVE IMPAIRMENT BY THEIR COGNITIVE STATUS

**DOI:** 10.1093/geroni/igae098.2677

**Published:** 2024-12-31

**Authors:** Mina Hwang, Yeji Hwang

**Affiliations:** Seoul National University, Seoul, Republic of Korea; Seoul National University, Seoul, Republic of Korea

## Abstract

Anxiety is common neuropsychiatric symptoms of dementia. While various factors can trigger anxiety in individuals with cognitive impairment, there is limited information available regarding whether these factors vary based on one’s cognitive status. Therefore, this study aimed to examine predictors of anxiety in older adults with cognitive impairment by their cognitive status. Using data from the National Health and Aging Trends Study (NHATS) Round 12, 1,040 older adults with probable or possible dementia were analyzed. Participants were categorized into possible (n=508, 48.8%) and probable dementia (n=532, 51.2%) groups. For data analyses, descriptive analyses, chi square tests, and logistic regression analyses were conducted. In the group of possible dementia, when participants had pain (OR=3.48, p < 0.001) or depression (OR=6.55, p< 0.001), they were more likely to present anxiety. In the group of probable dementia, when participants had pain (OR=3.54, p < 0.001), depression (OR=7.84, p< 0.001), low community cohesion (OR=1.59, 0=0.044), or were dependent on activities of daily living (OR=2.42, p< 0.001), they were more likely to present anxiety. This study found that while pain and depression were related to anxiety in both probable and possible dementia groups, factors such as dependency on activities of daily living or community cohesion were only related to anxiety in probable dementia group. When persons living with cognitive impairment present anxiety, healthcare professionals and caregivers should consider those triggers to reduce their anxiety.

